# Supplementary material for: Functional Versatility of the Human 2-Oxoadipate Dehydrogenase in the L-Lysine Degradation Pathway toward Its Non-Cognate Substrate 2-Oxopimelic Acid
Source: Int J Mol Sci. 2022 Jul 26;23(15):8213. doi: 10.3390/ijms23158213 (PMC9367764; doi:10.3390/ijms23158213)
Supplement: Supplementary file 1 [file ijms-23-08213-s001.zip › ijms-1808238-supplementary.pdf]

Supplementary Materials

SI Results and Discussion

Chemoenzymatic application of E1a as well as of E1o and E. coli E1o via enzymatic carboligation reactions

The utility of ThDP-dependent enzymes in asymmetric synthesis of α-hydroxy ketones as precursors for fine chemicals in the pharmaceutical industry has been extensively studied and reviewed over the recent decades [1-9]. More recent application of the ThDP-dependent carboligases is found in biocatalytic cascade reactions (so called biocatalytic cascade strategy) [10,11]. Considering the broad substrate specificity of the E1a, it could be effectively employed in chemoenzymatic synthesis of 2-oxohexandioic, 2-oxoheptandioic, 2-oxohexanoic and 2-oxoheptanoic acids as presented in Scheme S1. In general, the title compounds could be synthesized from the one-carbon shorter homologue via a three steps synthetic route. *The key to the novel application is the reaction of the 2-oxo acid with the first E1 component* (E1o or E1a) of the corresponding 2-oxo acid dehydrogenase complex (all E1's are ThDP-dependent enzymes), shortening the carbon chain by one carbon atom *via* decarboxylation to the E1a-ThDP-bound enamine intermediate, which then undergoes a carboligation reaction with glyoxylate, increasing the chain length by two carbon atoms, yielding a 2-hydroxy-3-oxoalkanoic acid. *In the second step,* the 3-oxo group could be reduced by $NaBH_4$ yielding a 2,3-dihydroxy-alkanoic acid; the latter in *the third step* is dehydrated to an alkenoic acid, expected to be formed by initial abstraction of the C2α-proton, followed by loss of the 3-hydroxyl group. The dehydration creates an enol that on spontaneous ketonization yields the 2-oxo acid product. Two recombinant enzymes, the human E1o and E1a in the authors' laboratories, and the *E. coli* E1o earlier engineered in the authors' laboratories [12, 13] provide the enzymes required for the first step. Plausible extension of the method includes the following:

(i) Creation of a large variety of 3-(alkyl, aryl)-amino-2-hydroxy-alkanoic mono and diacids via reductive amination of the ketone produced in step one.

(ii) Conversion of novel 2-oxo acids to the CoA thiolesters with one fewer CH_2 group using the enzymes in the authors' laboratories.

(iii) Creation of 2-oxoadipic acid and 2-oxopimelic acid semialdehyde intermediates (afforded by the ability of the E1a (E1o)-ThDP-bound enamine intermediate to undergo carboligation reactions with both

glyoxylate and its ethyl ester, rendering it DIBALH reducible) which can be used for further chain elongation via aldol-type condensation leading to both even and odd- carbon fatty acid derivatives/mimics. We further emphasize that the initial enzymatic carboligation leads to the introduction of a chiral α-ketol (observed and its stereochemistry assigned according to a well-characterized CD band at 278 nm), while chiral reducing agents are available for the reductive amination reactions, thereby creating the possibility of generating all four diastereomeric products, so crucial in pharmaceutical research for identification of the active stereoisomer.

Homologation of 2-oxo acids via carboligation and its synthetic applications

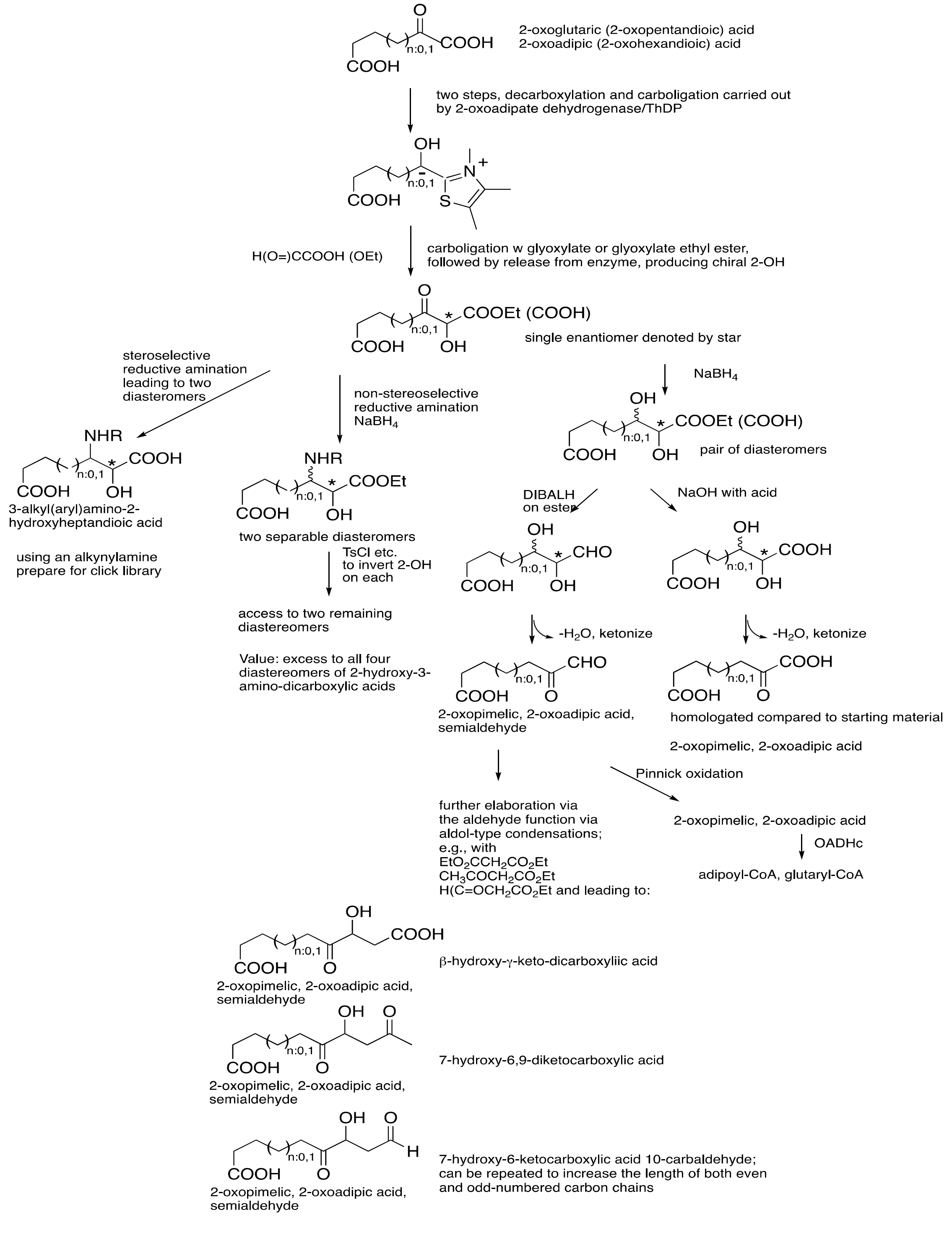

Scheme S1. Potential chemoenzymatic application of E1a *via* carboligation

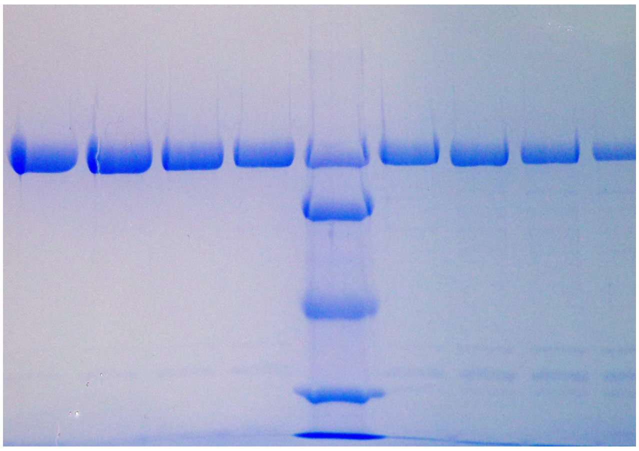

Figure S1. SDS-PAGE of the fractions of human E1a eluted after affinity chromatography using a Ni column.

SI References

[1] Jordan F. Current mechanistic understanding of thiamin diphosphate-dependent enzymatic reactions. *Nat. Prod. Rep*. **2003**, *20*, 184-201.

[2] Baykal, A.; Chakraborty, S.; Dodoo, A.; Jordan, F. Synthesis with good enantiomeric excess of both enantiomers of α-ketols and acetolactates by two thiamin diphosphate-dependent decarboxylases. *Bioorg. Chem.* **2006**, *34*, 380-393.

[3] Brandt G.S.; Kneen, M.M.; Petsko, G.A.; Ringe, D.; McLeish, M.J. Active -site engineering of benzaldehyde lyase shows that point mutation can confer both new reactivity and susceptibility to mechanism-based inhibition. *J. Am. Chem. Soc*. **2010**, *132*, 438-439.

[4] Meyer, D.; Walter, L.; Kolter,G.; Pohl, M.; Müller, M.; Tittmann, K. Conversion of pyruvate decarboxylase into enantioselective carboligase with biosynthetic potential. *J. Am. Chem. Soc.* **2011**, *133*, 3609-3616.

[5] Hailes, H.C.; Rother, D.; Müller, M.;Westphal, R.; Ward, J.M.; Pleiss, J.; Vogel, C.; Pohl, M. Engineering stereoselectivity of ThDP-dependent enzymes. *FEBS J.* **2013**, *280,* 6374-6394.

[6] Müller, M.; Sprenger, G.A.; Pohl, M. C-C bond formation using ThDP-dependent lyases. *Curr. Opin. Chem. Biol.* **2013**, *17*, 261-270.

[7] Beigi, M.; Waltzer, S.; Zarei, A.; Eggeling, L.; Sprenger, G.A.; Müller, M. TCA cycle involved enzymes SucA and Kgd, as well as MenD: efficient biocatalysts for asymmetric C-C bond formation. *Org. Lett.* **2013**, *15*, 452-455.

[8] Kasparyan, E.; Richter, M.; Dresen, C.; Walter, L.S.; Fuchs, G.; Leeper, F.J.; Wacker, T.; Andrade, S.L.A.; Kolter, G.; Pohl, M.; Müller, M. Asymmetric Stetter reactions catalyzed by thiamine diphosphate-dependent enzymes. *Appl. Microbiol. Biotechnol*. **2014**, *98*, 9681-9690.

[9] Baraibar, A.G.; Lieres, E.; Wiechert,W.; Pohl, M.; Rother, D. Effective production of (*S*)-α-hydroxy ketones: a reaction engineering approach. *Top Catal*. **2014**, *57*, 401-411.

[10] Schapfl, M.; Baier, S.; Fries, A.; Ferlaino, S.; Waltzer, S.; Müller, M.; Sprenger, G.A. Extended substrate range of thiamin diphosphate-dependent MenD enzyme by coupling of two C-C-bonding reactions. *Applied Microbiol Biotechn.* **2018**, *102*, 8359-8372.

[11] Kulig, J.; Sehl, T.; Mackfeld, U.; Wiechert, W.; Pohl, M.; Rother, D. An Enzymatic 2-step cofactor and co-product recycling cascade towards a chiral 1,2-diol. Part I: cascade design. *Adv. Synth. Catal.* **2019**, *361*, 2607-2615.

[12] Shim, D.J.; Nemeria, N.S.; Balakrishnan, A.; Patel, H.; Song, J.; Jordan,F.; Farinas E.T. Assignment of function to Histidines 260 and 298 by engineering the E1 component of the *Escherichia coli* 2-oxoglutarate dehydrogenase complex; substitutions that lead to acceptance of substrates lacking the 5-carboxyl group. *Biochemistry* **2011**, *50*, 7705-7709.

[13] Chakraborty, J.; Nemeria, N.S.; Zhang, X.; Nareddy, P.R.; Szostak, M.; Farinas, E.; Jordan, F. Engineering 2-oxoglutarate dehydrogenase to a 2-oxo aliphatic dehydrogenase complex by optimizing consecutive components. *AIChE J.* **2019**, *66* (3): e16769.
